# Nitrogen allocation among leaves and roots mediates the interaction between plant life history trade-off and density dependence

**DOI:** 10.3389/fpls.2025.1549801

**Published:** 2025-03-12

**Authors:** Junkang Cheng, Shixiao Yu

**Affiliations:** State Key Laboratory of Biocontrol/School of Life Sciences, Sun Yat-sen University, Guangzhou, China

**Keywords:** conspecific density dependence, functional traits, intraspecific variation, life history strategy, nutrient allocation

## Abstract

**Introduction:**

Carbon, nitrogen and phosphorus, as the basic components of plants, determine plant growth and adaptation strategies, while there are certain differences in nutrient allocation among different plant organs. However, little is known about the manner in which resource allocation mediates the plant life history strategy.

**Methods:**

Here, we collected three census field survey datasets from the Heishiding 50-ha dynamic plot showing functional traits and nutrient allocation among leaves and roots (⍺_nutrient_) from 92 woody species to determine the relationship between nutrient allocation and the plant life history strategy.

**Results:**

Carbon allocation ⍺_carbon_ was mainly determined by intraspecific variation while nitrogen allocation ⍺_nitrogen_ and phosphorus allocation ⍺_phosphorus_ was determined by interspecific variation. Species allocating more nitrogen to leaves showed greater resource acquisition traits, while species allocating more nitrogen to roots showed greater resource conservation traits. We found a trade-off between the plant relative growth rate and conspecific density dependence; fast-growing species showed higher mortality with conspecific neighbors but tended to allocate more nitrogen to leaves rather than roots.

**Discussion:**

Our study revealed interspecific variation in nutrient allocation among leaves and roots as well as their relationship with functional traits and the plant life history strategy.

## 1 Introduction

Coexistence in diverse ecological communities has been thought to be due to a life history trade-off involving resource allocation. The interspecific growth–mortality trade-off is widely accepted in forest ecosystems, which clarifies the relationship between plant growth and survival ability, and fast-growing species always take the risk of high mortality (Loehle, 1988; Wright et al., 2010; Russo et al., 2021). An increasing number of studies have combined the interspecific growth–mortality trade-off and multiple coexistence mechanisms to explain different plant performances among species, such as conspecific density negative dependence (CNDD). CNDD indicates a decrease in the survival probability of seedlings or saplings with increased conspecific neighbor density due to strong intraspecific competition and host-specific natural enemies (Janzen, 1970; Connell, 1971). Many studies have indicated the interspecific variation on the sensitivity to conspecific neighbors, whereas the life history strategy may explain the variation—fast-growing and shade-intolerant species showed higher mortality with conspecific neighbors (Zhu et al., 2018; Brown et al., 2020).

Trait-based ecology combines the individual organisms’ performance with community structure and function; it is used to explore the insight of plant life history strategies. Functional traits are defined as morpho-physio-phenological traits that indirectly impact fitness via their effects on growth, reproduction, and survival, which are the three components of individual performance (Violle et al., 2007). The proposed plant economics spectrum (PES) reveals the resource allocation trade-off among plant functional traits (Wright et al., 2004; Adler et al., 2014). Tissue construction for functional traits varies among species, constrained by a trade-off between resource acquisition and conservation, explaining the interspecific variation of the life history strategy across resource gradients (Reich, 2014; De la Riva et al., 2021). Higher resource acquisition traits, such as species leaf area and leaf nitrogen (N) content, indicate that species showed a higher growth rate under a resource-plenty environment, whereas species with higher resource conservation traits, such as tissue dry mass content and wood density, show greater defense and survival when resources are scarce (Wright et al., 2010; Adler et al., 2014; Song et al., 2021). Through the understanding of functional traits among PES, ecologists found factors that mediate the interspecific variation in plant susceptibility to conspecific neighborhoods. Lebrija-Trejos et al. (2016) revealed that the tolerance of seedling CNDD enhanced recruit survival, which was greater with a larger seed mass in slow-growing and well-defended species.

Resource allocation among plant organs, including the allocation of biomass and nutrients, showed an important reflection of plant growth and adaptation strategies (Kerkhoff et al., 2006; Reich et al., 2008; Sardans and Penuelas, 2013; Umaña et al., 2021). Carbon (C), nitrogen (N), and phosphorus (P) are the fundamental elements associated with the chemical composition of living organisms, and their concentrations regulate plant growth and directly reflect plant adaption to environmental changes (Elser et al., 2007; Hu et al., 2021). Therefore, it indicates an assignment to explore whether the allocation pattern of C, N, and P among different plant organs plays a crucial role in mediating the plant life history strategy (Reich et al., 2008; Minden and Kleyer, 2014; Zhao et al., 2016). Through information on leaf, stem, and root mass fractions of 97 species from a tropical forest, Umaña et al. (2021) demonstrated that the limited soil N resulted in a high mass allocation in leaves, whereas the opposite trend was observed in response to limited soil P. Yan et al. (2016) determined the N and P concentrations between stems and leaves of 335 woody species from 12 forests and found that as latitude increased, the scaling exponents for N and P significantly decreased between leaves and stems.

However, considerable research has only focused on functional groups at the individual and community levels under different abiotic conditions (Aoyagi and Kitayama, 2016; Freschet et al., 2018; Hu et al., 2021; Liang et al., 2022), ignoring the interspecific variation. Thus, little is known about the relationship between resource allocation among organs and plant life history strategies. Previous studies have primarily explored the role that functional traits play in plant growth and adaptation strategies (Adler et al., 2014; Umaña et al., 2023; Fajardo et al., 2024; Lam et al., 2024). However, the variation in resource allocation may prove more insight in understanding the mechanism of the plant life history trade-off, while C, N, and P have been proven to play different roles among different plant organs. In foliar, C, N, and P are required as important elements for photosynthesis and other metabolism processes (Ahanger et al., 2019; Mu and Chen, 2021; Suriyagoda et al., 2023; Xue et al., 2023), while the maximum photosynthetic rate is positively correlated with foliar N and P (Reich et al., 2009; Tosens et al., 2016; Evans and Clarke, 2019). In non-photosynthesis organs, such as stems and roots, they may serve as components of proteins or cell walls for defense (Onoda et al., 2004; Mur et al., 2017; Zipfel and Oldroyd, 2017). So the difference of element allocation among different organs between species may determine the life-history strategy and plant performance.

In this study, we determined the functional traits and nutrient content for leaves and roots of 92 woody species, combined three census field survey datasets from a 50-ha subtropical forest plot, and explored the role of nutrient allocation among leaves and roots in mediating the interspecific life history trade-off. Here, we asked three specific questions: 1) Does interspecific variation play an important role in nutrient allocation among leaves and roots? 2) Does nutrient allocation among different organs determine the interspecific PES? 3) Does nutrient allocation among different organs determine the trade-off between the growth rate and density-dependent survival?

## 2 Materials and methods

### 2.1 Study site

This study was carried out in the Heishiding 50-ha dynamic plot of the subtropical evergreen broad-leaved forest located in the Heishiding Nature Reserve (Guangdong Province, southern China; 111°53′ E, 23°27′ N, 150–927 m a.s.l.). The mean annual precipitation is 1,744 mm, occurring mainly between April and September (79% of annual rainfall). The mean annual temperature is 19.6°C, and the mean monthly temperature ranges from 10.6°C in January to 28.4°C in July.

The dynamic plot was established in 2011, where all trees with the diameter at breast height (DBH) ≥1 cm were tagged, identified, measured, and mapped using the standard CTFS-ForestGEO protocol. Censuses were conducted every 5 years. According to the last census (2021), the dominant species in the investigated plot belonged to the Fagaceae and Lauraceae families.

### 2.2 Field sampling

Species with more than 50 sapling (1 cm < DBH ≤ 5 cm) individuals in the dynamic plot were chosen as study objects. This cutoff was consistent with previous studies, while the trade-off between growth and mortality was found strongest under the sapling stage (Peters, 2003; Condit et al., 2006; Wright et al., 2010; Zhu et al., 2018). This leaves 92 focal woody species (of 181 woody species present in the Heishiding 50-plot dynamic plot) from 51 genera and 30 families. We randomly selected eight individuals with similar size per species from the area, and the distance between each individual of the same species was more than 30 m. Leaves and fine roots were sampled for morphological traits and nutrient content measurements. Mature leaves under each selected sunlit individual were collected with a high branch cutter. We collected root samples according to the extension direction of the root system at the base of each target individual, with a spade to dig and separate the root system, while the root segments with diameter ≤2 mm were defined as fine roots. Sampling was carried out from March to July 2021, lasting for 4 months.

#### 2.2.1 Nutrient allocation among leaves and roots

Healthy mature leaves and fine root branches were dried in an oven at 75°C for at least 72 h, and then ground and passed through a 100-mesh sieve. The total N content of the leaves and fine roots was determined using a Kjeldahl apparatus (Kirk, 1950); a 0.25-g ground sample was digested with 5 mL of concentrated H_2_SO_4_ at 380°C using Na_2_SO_4_ and CuSO_4_ as catalysts until the solution became clear. The total P content was determined using phosphorus–vanadate–molybdate blue colorimetry (Taussky and Shorr, 1953); the sample was dissolved in HNO_3_. NH_4_VO_3_ and (NH_4_)_2_MoO_4_ were added, and the absorbance of the generated phosphorus–vanadate–molybdate blue was measured using a spectrophotometer to calculate the phosphorus content. The total C content was determined using the oxidizing organic matter method with K_2_Cr_2_O_7_ in H_2_SO_4_ (96%) for 30 min (Perez-Harguindeguy et al., 2013). Nutrient allocation among leaves and roots was calculated as the stoichiometric scaling relationship of leaf N (or P or C) and root N (or P or C) using all original data of the nutrient concentration from individual samples:


$$\alpha_{nutrient}=\;\frac{leaf\;nutrient\;content}{root\;nutrient\;content}$$

#### 2.2.2 Morphological trait measurement

We selected three to five healthy, complete, mature leaves from each individual, weighed them to determine their fresh mass, and determined the leaf thickness (T, mm) with a micrometer. The leaves were scanned using a flatbed scanner (Epson V370, China), and the leaf area (LA, cm^2^) was determined with ImageJ (version 1.43u, USA). For scanning (Epson V700, USA), two to three fine root branches (diameter <2 mm) with intact terminal branch orders were cut and spread out in a water bath. WinRHIZO 2013e software (Regent Instrument Inc., Canada) was used to analyze the scanned images and obtain the average root diameter (DIAM, mm), number of root tips, root length, surface area, and volume. After drying in an oven at 75°C for 72 h, the leaf and root dry mass were determined.

The specific leaf area (SLA, cm^2^ g^−1^) was calculated by dividing the leaf area by its dry mass. The leaf dry mass content (LDMC, %) was calculated as the ratio of the leaf dry mass to its fresh mass. The root tissue density (RTD, g cm^−3^) was calculated as the ratio of root dry mass to its volume, assuming that the root was a cylinder. The specific root length (SRL, cm g^−1^) was calculated as the root length divided by its dry mass. The specific root area (SRA, cm^2^ g^−1^) was calculated as the root surface area based on its dry mass. The root branching intensity (RBI, tips cm^−1^) was calculated as the number of root tips in a certain root length.

#### 2.2.3 Calculation of growth rate and neighbor densities

We calculated the relative growth rate (RGR) of each sapling individual among the following 5-year census intervals: 2011–2016 and 2016–2021. The RGR was calculated as (dbh_t+1_ − dbh_t_)/time. For the 92 focal species, we used the 95-percentile relative growth rate (RGR_95_) as a proxy for growth under favorable conditions (Russo et al., 2021). Based on the RGR_95_, we divided species into three categories: slow-growing, median-growing, and fast-growing species (Wright et al., 2010) (**Supplementary Table S1**).

To test the density dependence effect on plant survival, we calculated the densities of conspecific and heterospecific neighbors for each focal individual. Conspecific and heterospecific densities were calculated as the sum of the basal area of all conspecific and heterospecific trees ≥ 1 cm dbh within a radius of 10 m at the start of the census interval in 2011 and 2016, as follows (Comita et al., 2010):


$$ConBA_{i}\;or\;HeterBA_{i}=\sum_{j=1}^{n}{\left(\frac{BA_{j}}{Dist_{ij}}\right)}^{\alpha }$$

where n is the number of neighbors within radius 10 m, *BA_j_* is the basal area of neighbor *j*, and *Dist_ij_* is the distance between focal sapling *i* and its neighbor *j*. To account for the potentially non-linear nature of local biotic interactions, we introduced exponent α, ranging from 0.1 to 1, and selected the α value with the minimum AIC (α = 0.6, **Supplementary Table S2**) (Detto et al., 2019). Shrub and liana individuals, which are monitored in the censuses, were included in the calculation of neighbor densities, but only tree saplings were included as focal individuals in the analysis.

### 2.3 Data analysis

For the normal distribution of data, the log value was determined for the traits and $\alpha_{nutrient}$ . All statistical analyses were performed using R-4.1.1 (R Core Team, 2021).

The “varcomp” function in the “ape” package (Paradis and Schliep, 2019) was used to calculate the variance components associated with the nested levels. This analysis assessed the variation in nutrient allocation among species (interspecific variation) and individuals (intraspecific variation). Blomberg’s K was used to test the phylogenetic signal of functional traits and nutrient allocation (Blomberg et al., 2003). Principal component analysis (PCA) was performed with the nine morphological traits (LA, SLA, LDMC, T, DIAM, RTD,RBI, SRA, SRL) using the “rda” function in the “vegan” package (Oksanen et al., 2018). To determine how well nutrient allocation predicted plant morphological traits, we generated univariate and multivariate phylogenetic generalized least squares (PGLS) models for $\alpha_{nutrient}$ and other traits by fitting a Brownian motion model for character evolution (Martínez-Vilalta et al., 2010) using the function “pgls” in the “caper” package (Orme, 2013).

To explore nutrient allocation-mediated density dependence survival, we generated hierarchical Bayesian models that allowed for variation in neighbor effects among species (Comita et al., 2010; Johnson et al., 2012; Song et al., 2021). In the individual-level regression, the survival (*p*) of an individual sapling (defined as the status at the ending year of both 5-year census intervals) was modeled as a function of the initial DBH, ConBA, and HeterBA, whereas the quadrats and plot census interval (*φ_q_*) of the sapling individuals were included as random effects to control for spatial variation and temporal autocorrelation, as follows:


$$surv_{isq}\;=Bernoulli(p_{sq})$$

$$logit(p_{sq})=\beta_{0s}+\beta_{1s}\cdot DBH_{s}+\beta_{2s}\cdot ConBA_{s}+\beta_{3s}\cdot HeterBA_{s}+\varphi_{q}$$

At the species level, the coefficients (*β_0–3_*) of each species were modeled as a function of nutrient allocation ( $\alpha_{nutrient}$ ), as follows:


$$\beta_{ms}\;∼\;N(\gamma_{m0}+\gamma_{m1}\cdot \alpha_{nutrient},\;\sigma_{m}^{2})$$

All individual- and species-level coefficients were assigned weakly informative priors. Cauchy (0, 5) was used as the prior of the regression coefficients and scale parameters. Bayesian inferences were performed using Stan (Stan Development Team, 2022) in R version 4.1.1 (R Core Team, 2021). We ran two independent chains with different initial values. All models were run for 20,000 iterations, within which there were 10,000 warm-ups. Convergence was ensured, and no transitions existed after warm-up in any of the Bayesian sampling chains.

## 3 Results

**Figure 1:**
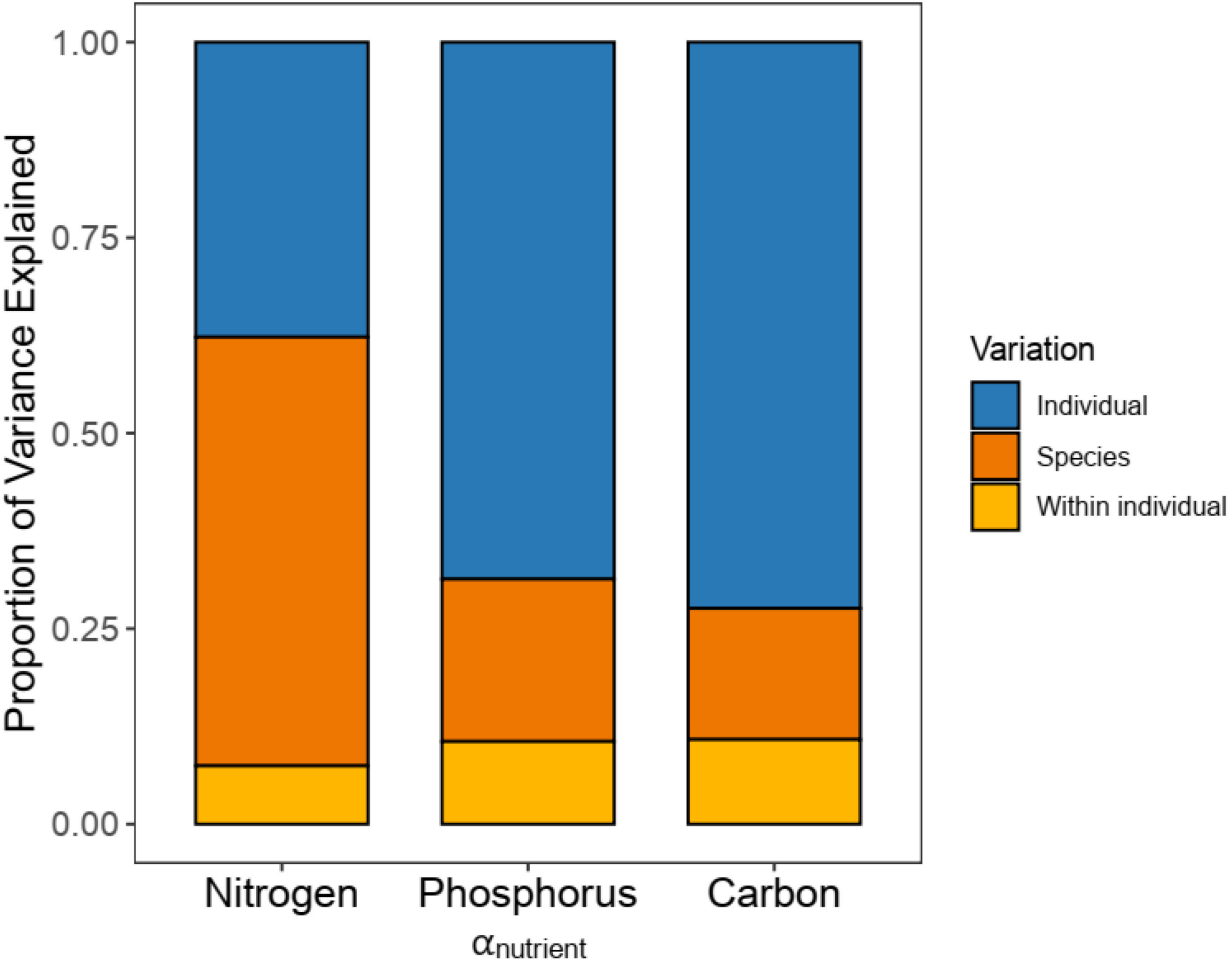
Variance partitioning of nutrient allocation between leaves and roots among 92 woody species across two levels (individual and species).

N and P allocation between leaves and roots (α_Nitrogen_ and α_Phosphorus_) varied among individuals (**Supplementary Figure S1**), whereas species explained 60.08% and 42.34% of the variation in α_Nitrogen_ and α_Phosphorus_, respectively (**Figure 1**). For α_Carbon_, there was less variation among individuals (CV = 14.01%), which was mostly explained by intraspecific variation. Correlation analysis of nutrient allocation showed a significant positive relationship between α_Nitrogen_ and α_Phosphorus_ (*P* ≤ 0.01), indicating that species allocating more N to leaves also allocated more P to leaves (**Supplementary Figure S2**).

The analysis of phylogenetic signals showed that except for LA and α_Carbon_, plant morphological traits and nutrient allocation were significantly affected by the plant’s evolutionary history in 92 species (**Supplementary Table S3**). The K-values of root traits (0.187–0.332) were higher than those of leaf traits (0.109–0.213), and those of N and P allocation between leaves and roots were 0.235 and 0.318, respectively.

**Figure 2:**
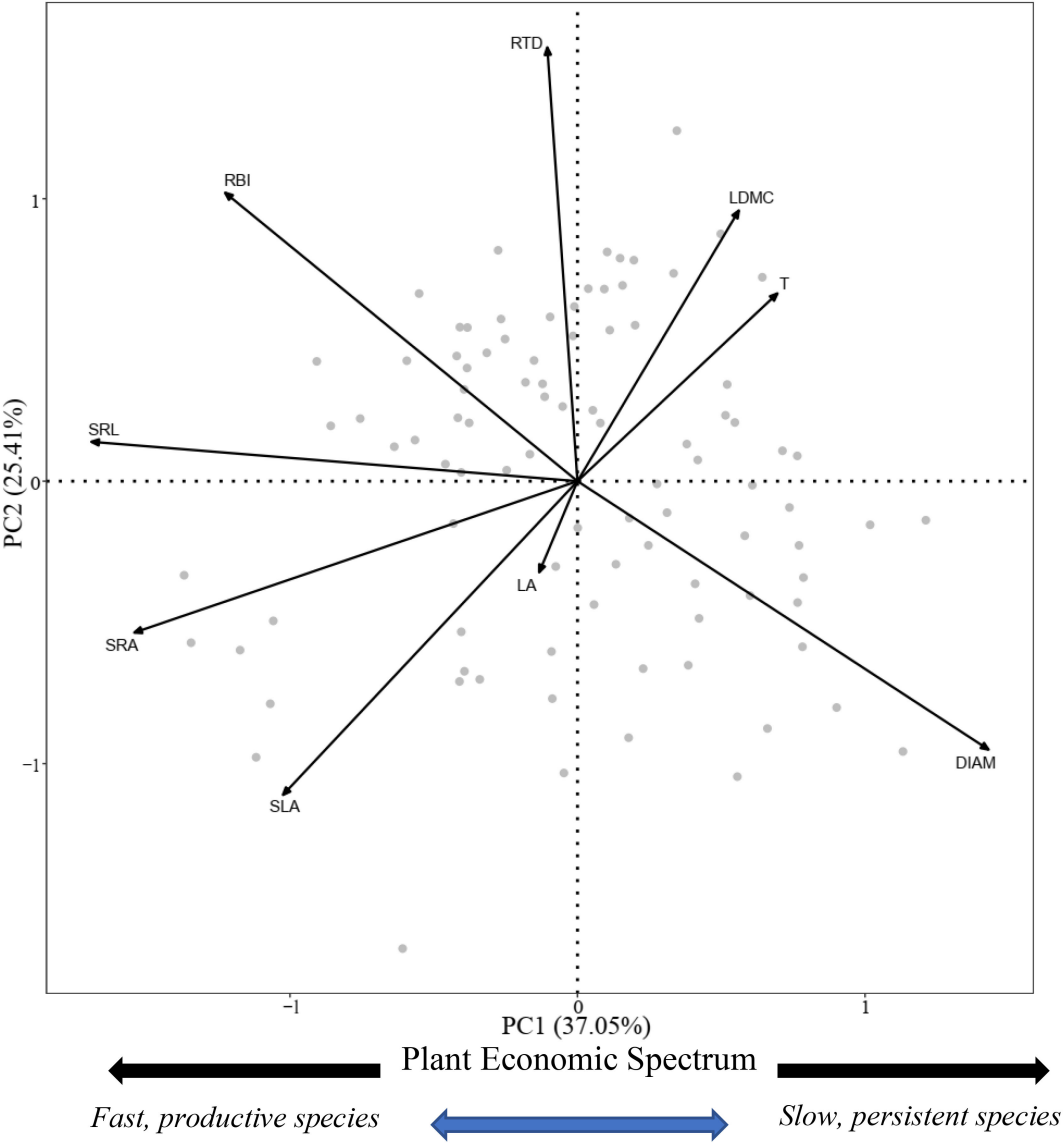
Plot of the first and second axes of the principal component analysis (PCA) performed with nine morphological traits and 92 woody species. The plant economic spectrum was determined following Reich (2014). LA, leaf area; SLA, specific leaf area; LDMC, leaf dry mass content; T, leaf thickness; DIAM, root diameter; RTD, root tissue density; SRA, specific root area; SRL, specific root length; RBI, root branch intensity.

PCA showed that covariation among nine plant morphological traits was represented by two independent dimensions, with a cumulative explanatory power of 62.46% of the variation (**Figure 2**). The variation within the first axis was defined as the PES. In this axis, LDMC, T, and DIAM had a positive association, whereas SLA, RBI, SRA, and SRL had a negative association. Following Reich (2014), we divided the nine morphological traits into two categories: LA, SLA, RBI, SRA, and SRL as resource acquisition traits; LDMC, T, DIAM, and RTD as resource conservation traits. Considering the significantly phylogenetic signal of traits and nutrient allocation, we used the PGLS model to analyze the relationship between resource acquisition traits and resource conservation traits. There was a significant negative relationship between the two categories (expect for RBI and RTD). According to univariate PGLS, SLA, SRA, and SRL showed a significant negative relationship with more than two resource conservation traits, whereas there were significant relationships between SRA and all resource conversation traits (**Table 1**). RBI had a significant negative relationship with DIAM and a positive relationship with RTD. However, LA showed no significant relationship with any of the resource conservation traits, which was consistent with the analysis of the phylogenetic signal that LA was not significantly affected by the species’ evolutionary history (**Supplementary Table S3**). Optimal multivariate PGLS showed that resource conversation traits were strong predictors of resource acquisition traits, except for LA, with R^2^ values ranging from 0.261 to 0.991 (**Table 2**). LDMC and T were the best predictors for SLA, whereas DIAM played an important role in predicting root resource acquisition traits. The strong relationship between resource acquisition and conservation traits showed the existence of a PES.

**Table 1:** Coefficient of univariate phylogenetic generalized least squares model (PGLS) for resource acquisition traits and resource conservation traits.

	LA	SLA	RBI	SRA	SRL
LDMC	−0.185	−0.554***	0.075	−0.228*	−0.108
T	0.085	−0.674***	−0.034	−0.304***	−0.259**
DIAM	0.081	−0.131	−0.491***	−0.675***	−0.944***
RTD	−0.188	−0.493***	0.210**	−0.601***	−0.124

*represents a significant relationship at *P* < 0.05; **represents a significant relationship at *P* < 0.01; ***represents a significant relationship at *P* < 0.001.

**Table 2:** Coefficient of optimal multivariate phylogenetic generalized least squares model (PGLS) for resource acquisition traits and resource conservation traits.

	LA	SLA	RBI	SRA	SRL
LDMC	−0.179	−0.535***			−0.021
T		−0.670***			−0.017
DIAM			−0.491***	−1.104***	−1.170***
RTD	−0.175	−0.143*		−0.968***	−0.497***
R^2^	0.070	0.812	0.261	0.982	0.991

*represents a significant relationship at *P* < 0.05; ***represents a significant relationship at *P* < 0.001.

Based on the phylogenetic signal of α_Nitrogen_ and α_Phosphorus_, we used PGLS to determine whether nutrient allocation between leaves and roots determined the PES. α_Nitrogen_ had a significant positive relationship with resource acquisition traits, including SLA, SRA, and SRL, whereas a significant negative relationship was found between α_Nitrogen_ and resource conservation traits, including LDMC, T, and DIAM (**Table 3**). As for α_Phosphorus_, there was also a significant positive relationship with SLA. There was no significant relationship between α_Carbon_ and the nine morphological traits (**Table 3**). The multivariate PGLS showed that α_Nitrogen_ was the best predictor for SLA, SRA, SRL, LDMC, T, and DIAM (**Table 4**). The PC1 score from the PCA analysis of nine morphological traits was used to represent the position of species in the PES. PC1 had significant negative relationships with α_Nitrogen_ and α_Phosphorus_ (*P* < 0.001), but α_Nitrogen_ was also the best predictor for PC1 (R^2^ = 0.154) in the multivariate PGLS analysis (**Tables 3**, **4**). Univariate and optimal multivariate PGLS models were also used to analyze the relationships between functional traits, nutrient allocation, and RGR_95_. SLA and α_Nitrogen_ showed a significant positive relationship with RGR_95_, whereas LDMC and T showed a significant negative relationship (**Table 5**).

**Table 3:** Coefficient of univariate phylogenetic generalized least squares models (PGLS) for functional traits and nutrient allocation in leaves and roots.

	Resource acquisition traits					Resource conservation traits				PC1
	LA	SLA	RBI	SRA	SRL	LDMC	T	DIAM	RTD	
α_Nitrogen_	−0.107	0.648***	0.091	0.320**	0.315**	−0.333*	−0.441***	−0.304**	0.110	−0.632***
α_Phosphorus_	0.019	0.444***	0.113	0.121	0.085	−0.096	−0.140	−0.181	0.214*	−0.342*
α_Carbon_	0.030	−0.002	−0.035	0.054	0.007	0.126	0.002	0.038	−0.108	0.167

*represents a significant relationship at *P* < 0.05; **represents a significant relationship at *P* < 0.01; ***represents a significant relationship at *P* < 0.001.

**Table 4:** Coefficient of optimal multivariate phylogenetic generalized least squares models (PGLS) for functional traits and nutrient allocation in leaves and roots.

	Resource acquisition traits					Resource conservation traits				PC1
	LA	SLA	RBI	SRA	SRL	LDMC	T	DIAM	RTD	
α_Nitrogen_	−0.107	0.648***		0.320**	0.315**	−0.333*	−0.441***	−0.304**		−0.632***
α_Phosphorus_			0.113						0.214*	
α_Carbon_										
R^2^	0.001	0.199	0.008	0.063	0.074	0.057	0.084	0.081	0.033	0.154

*represents a significant relationship at *P* < 0.05; **represents a significant relationship at *P* < 0.01; ***represents a significant relationship at *P* < 0.001.

**Table 5:** Univariate and optimal multivariate phylogenetic generalized least squares models (PGLS) for RGR_95_, functional traits, and nutrient allocation in leaves and roots.

	RGR_95_	
	Univariate PGLS	Multivariate PGLS
LA	0.128	
SLA	0.213*	
RBI	0.043	
SRA	0.181	
SRL	0.180	
LDMC	−0.247*	−0.211
T	−0.199*	−0.219
DIAM	−0.107	
RTD	−0.159	
R^2^		0.068
α_Nitrogen_	0.289*	0.505**
α_Phosphorus_	0.001	-0.298
α_Carbon_	0.053	
R^2^		0.062

*represents a significant relationship at *P* < 0.05; **represents a significant at *P* < 0.01.

**Figure 3:**
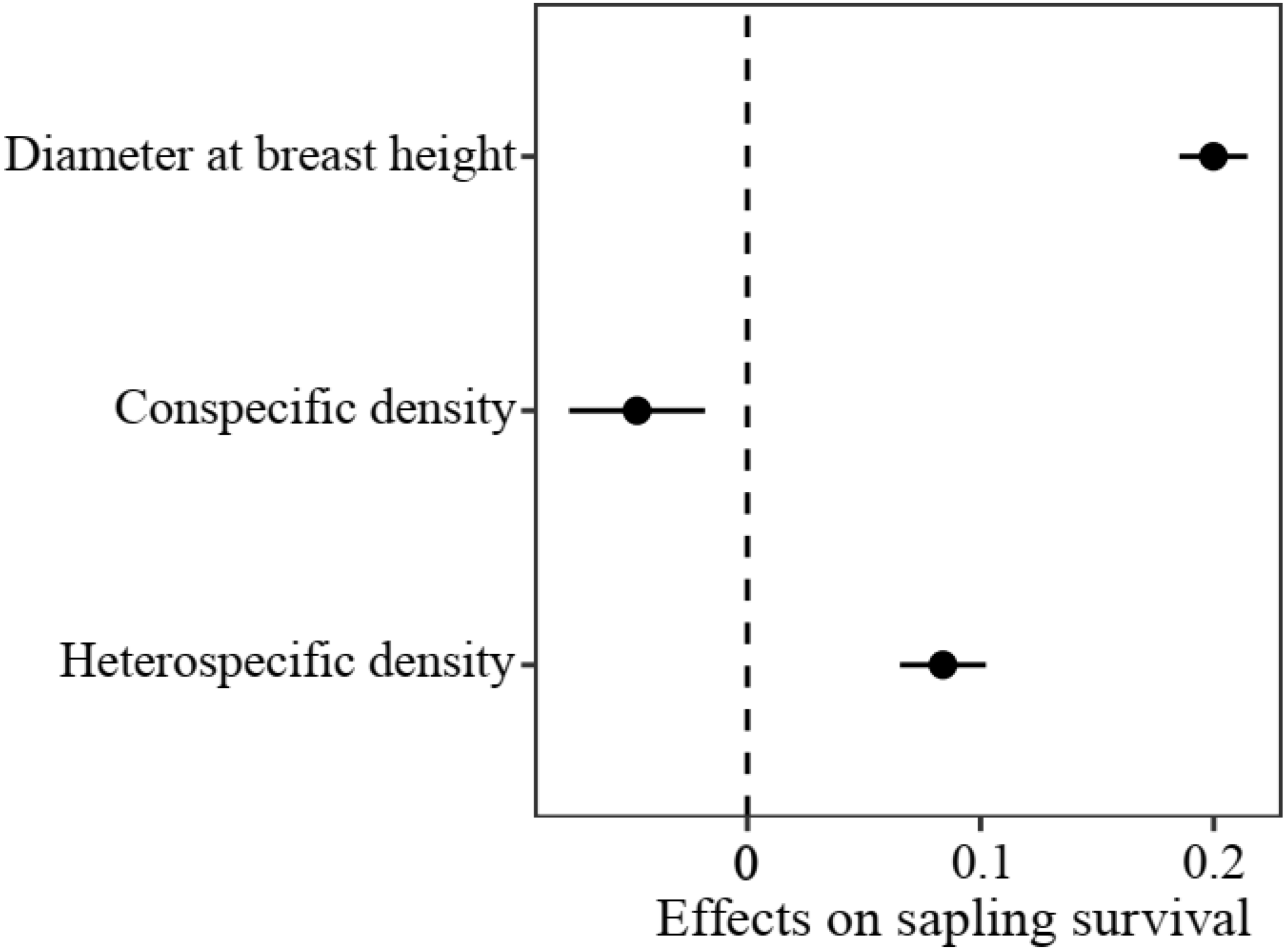
Initial plant size- and density-dependent effects on sapling survival at the community level. Values represent regression coefficients, and bars represent 95% confidence intervals. Solid circles indicate values that are significantly different from 0 (*P* < 0.05).

**Figure 4:**
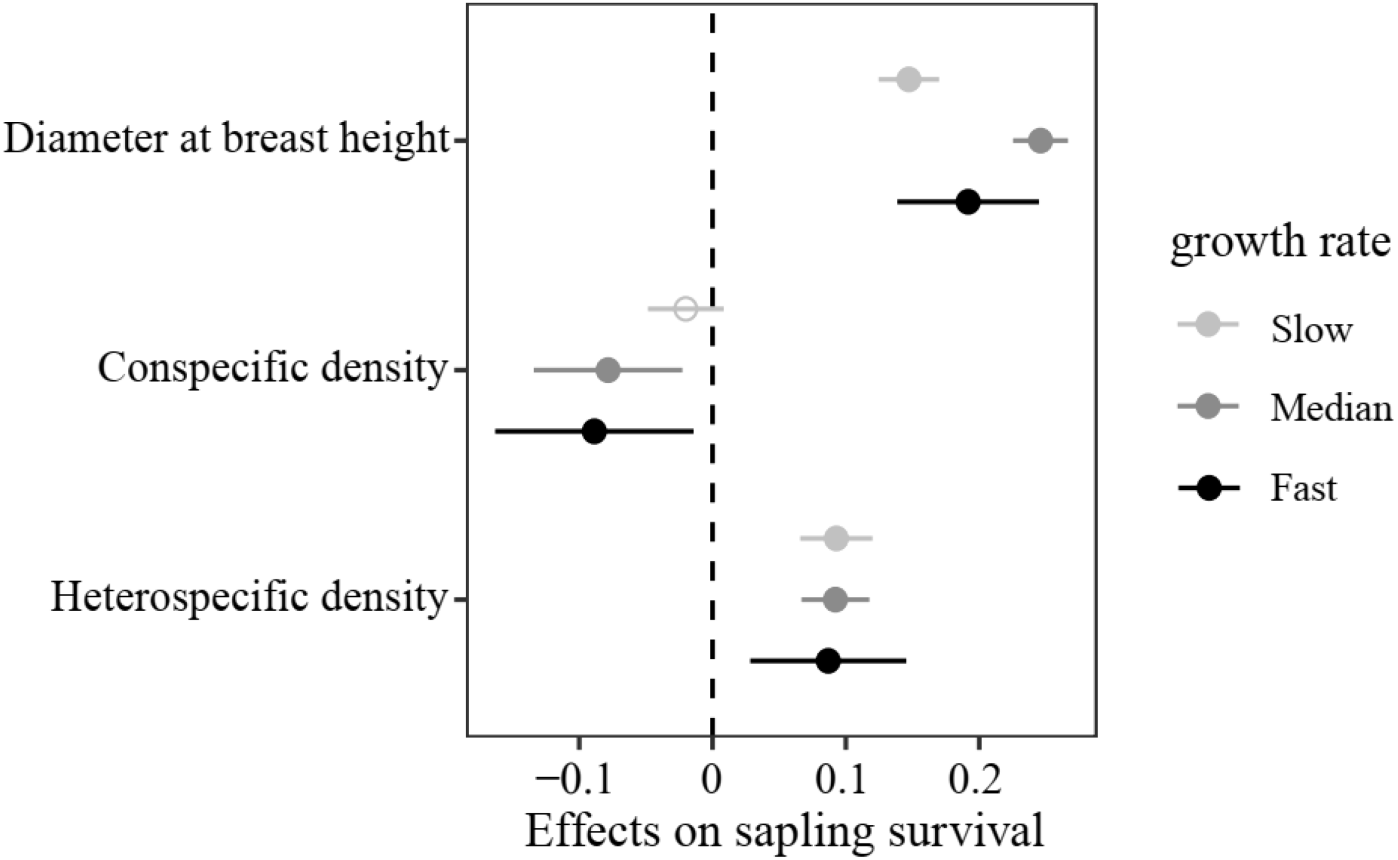
Initial plant size- and density-dependent effects on sapling survival among different species growth rates. Values represent regression coefficients, and bars represent 95% confidence intervals. Bold solid circles indicate values that are significantly different from zero (*P* < 0.05), and hollow circles indicate non-significant differences from zero.

**Figure 5:**
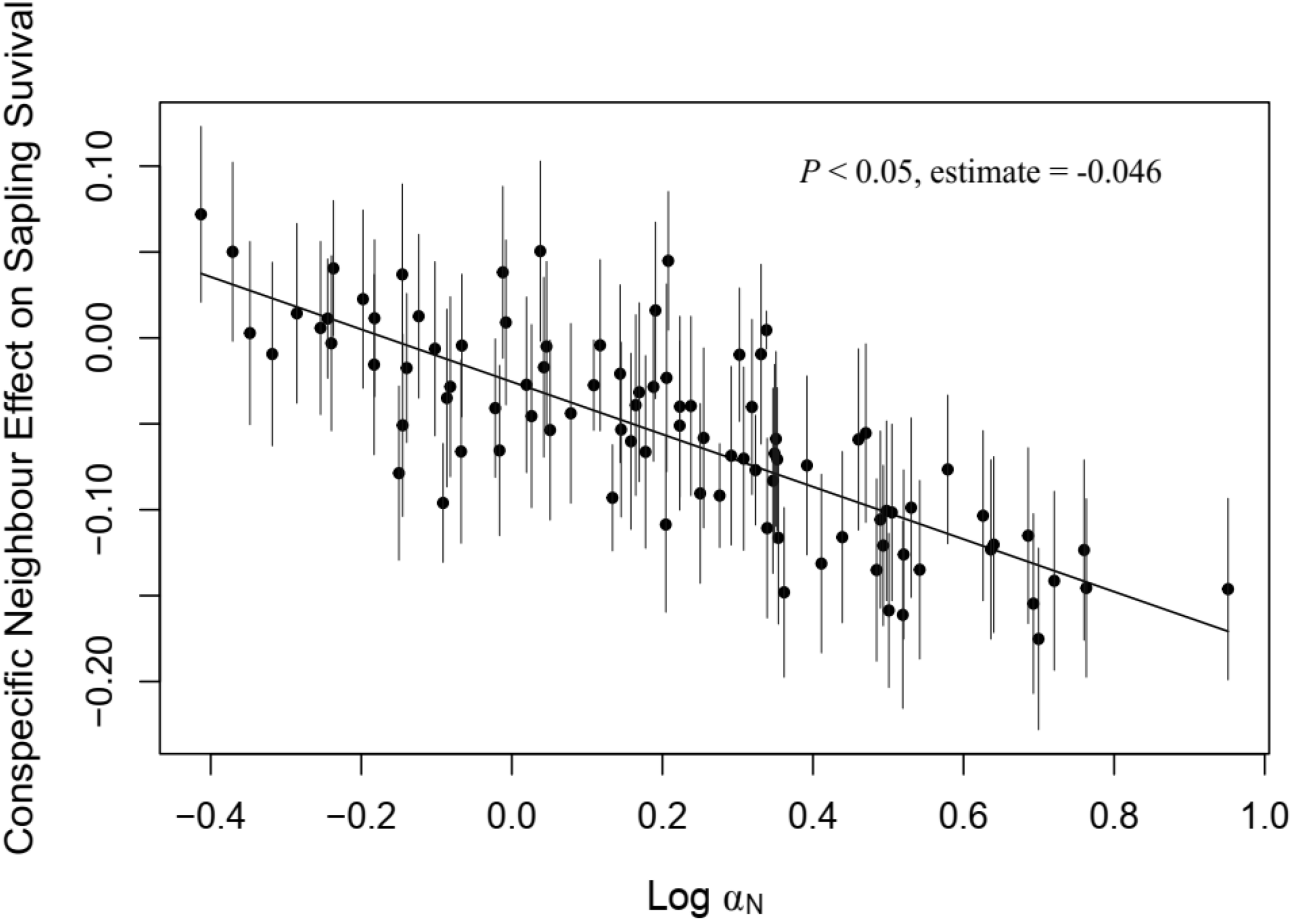
Relationship between nitrogen allocation in leaves and roots and species conspecific neighbor effects on sapling survival. Bars represent 95% confidence intervals. A solid line indicates a significant relationship (*P* < 0.05).

In the sapling stage, conspecific neighbor density showed a significant negative effect on the probability of survival, whereas DBH and heterospecific neighbor density were significantly positively associated with individual survival (**Figure 3**). Additionally, we found significant variation in the strength of CNDD among tree species (**Supplementary Figure S3**). To determine whether there was a trade-off between the tree growth rate and strength of CNDD under the sapling stage in the Heishiding 50 ha dynamic plot, we divided the species into three groups—slow-growing, median-growing, and fast-growing species—according to the RGR_95_ of each species. The strength of CNDD tended to be stronger with the increase in species RGR_95_ (**Figure 4**). The conspecific neighbor density showed no significant effect on sapling survival among slow-growing species (µ = −0.020; *P* = 0.168), whereas the significant negative effect of conspecific neighbor density on fast-growing species (µ = −0.089; *P* = 0.020) was stronger than that on median-growing species (µ = −0.078; *P* = 0.006). These results indicate a trade-off between species growth rate and the strength of CNDD at the sapling stage, whereas species growing faster have a stronger negative effect of CNDD. Using the hierarchical Bayesian models, we found that α_Nitrogen_ showed a significant negative relationship with the coefficients of conspecific neighbor effects, indicating that species allocating more N to leaves have a stronger CNDD (**Figure 5**). As for P and C, α_Phosphorus_ and α_Carbon_ also showed a negative relationship with the coefficient neighbor effects (**Supplementary Figure S4**).

## 4 Discussion

Our study determined the morphological traits and the C, N, and P contents of 92 woody species from a 50-ha subtropical forest plot and combined three census field survey datasets to explore the role of nutrient allocation between leaves and roots in the plant life history strategy and conspecific density dependence. We found that interspecific variation played an important role in N and P allocation between leaves and roots. α_nitrogen_ and α_phosphorus_ as well as plant morphological traits also had significant phylogenetic signals. N allocation between leaves and roots mediated the PES. Species with higher α_nitrogen_ (allocated more N in leaves) showed higher resource acquisition traits, such as SLA, SRA, and SRL, whereas species allocating more N to the roots showed higher resource conservation traits, such as LDMC, T, and DIAM. There was an interaction between the life history strategy and conspecific density dependence; fast-growing species always experienced greater conspecific negative effects. Nutrient allocation also played an important role in this interaction; species allocating more N to the leaves tended to be fast-growing and more sensitive to conspecific neighbors.

The study of concentrated resource allocation among plant organs remains rare and contradictory. Some studies have indicated obvious scaling relationships in resource allocation (biomass and nutrient) between above- and belowground parts (Zhao et al., 2020; Hu et al., 2021). However, more studies have reported significant variation in resource allocation among different abiotic conditions and plant lifeforms (Kerkhoff et al., 2006; Yan et al., 2016; Rao et al., 2020; Aoyagi et al., 2023). In our study, obvious interspecific variation was observed in N and P allocation between leaves and roots (α_Nitrogen_ and α_Phosphorus_) for 92 woody species at the sapling stage (**Figure 1**). As the fundamental elements associated with the chemical components of living organisms, the variation in N and P allocation may reflect the plant life history strategy and adaption strategy to the environment (Ågren, 2004; Elser et al., 2007). Moreover, we found a significant phylogenetic signal in α_Nitrogen_ and α_Phosphorus_ (**Supplementary Table S3**). Phylogeny plays an important role in community ecology by providing information about evolutionary relationships among species (Graves and Gotelli, 1993; Losos, 1996; Li et al., 2017). Our results revealed a relationship between N and P allocation among different organs and phylogenetic histories.

Significant phylogenetic signals for functional traits were also found among the 92 subtropical species in our study (**Supplementary Table S3**), consistent with previous studies (Webb et al., 2002; Donoghue, 2008; Li et al., 2017). The PGLS model was used in this study to avoid phylogenetic dependency between plant species due to a shared evolutionary history (Gerz et al., 2018; Shen et al., 2019). The resource acquisition traits were predictable from resource conservation traits, especially SLA, SRA, and SRL (**Tables 1**, **2**). Wright et al. (2004) proposed that leaf mass per area, which reflects the dry mass cost of deploying new leaf area, was important for leaf economics. Kraft et al. (2015) collected 222 species and reported strong relationships between functional traits and the plant life history, whereas SLA, as a resource acquisition trait, showed a significant relationship with plant growth, survival, and fecundity. However, for root functional traits, Bergmann et al. (2020) defined SRL on the resource collaboration axis, indicating the degree to which plants rely on the roots rather than their mycorrhizal partners. In our study, AM species accounted for the majority, whereas the nutrient foraging ability of AM species depended more on the variation of root morphology traits (Chen et al., 2016). Therefore, in our study, SRL played an important role in resource acquisition traits.

The growth–mortality trade-off reveals that species with fast growth rates and great resource acquisition traits always take the risk of high mortality (Wright et al., 2010; Russo et al., 2021). In our study, it was found that fast-growing species experienced higher negative conspecific density dependence compared with slow-growing species (**Figure 4**), consistent with previous studies (Zhu et al., 2018; Brown et al., 2020). The relationship between RGR and CNDD is most likely the result of differences in the allocation of limited resources. Several experiments have demonstrated that fast-growing, shade-intolerant species are more susceptible to herbivores and pathogens, whereas slow-growing species tend to allocate more resources to storage and defense (Kobe, 1997; Reich et al., 1998; Queenborough et al., 2013). Therefore, many studies have combined niche partitioning and CNDD to explain species coexistence and community assembly (Zhu et al., 2018; Brown et al., 2020; Song et al., 2021).

N and P are essential limiting resources for the primary productivity of terrestrial plants, whereas the content and allocation of these elements always plays an important role in plant growth and the life history strategy (LeBauer and Treseder, 2008; Vitousek et al., 2010; Yuan and Chen, 2012). In our study, the allocation of N rather than P among leaves and roots determined plant functional traits (**Table 4**) and the life history strategy (**Table 5**; **Figure 5**). N is a major component of proteins, nucleic acids, phospholipids, and chlorophyll in plant bodies, playing an important role in plant metabolism and plant growth promotion. N plays different roles in photosynthetic and non-photosynthetic organs. In leaves, the specific leaf N content positively affects photosynthesis (Allison et al., 1997; Mu and Chen, 2021). Rogers et al. (2017) combined the leaf N content and dry mass per area to determine a useful method for parameterizing photosynthesis. The relationship between leaf N and photosynthesis is partly related to N partitioning in photosynthetic enzymes, pigment content, and the size, number, and composition of chloroplasts (Marchiori et al., 2014; Li et al., 2015; Evans and Clarke, 2019). Species allocating more N in photosynthesis may tend to enhance their ability on light acquisition and strengthen the photosynthetic efficiency, which led to a fast-growing strategy. However, few studies have focused on N investment in non-photosynthetic organs, such as stems and roots. Although Bergmann et al. (2020) proposed the root economics space, the root N content indicated a conservation gradient with higher resource acquisition ability. However, in stems and roots, cell walls account for the bulk of the dry mass, whereas N is an important component of proteins (Onoda et al., 2004; Xue et al., 2023; Griffin-Nolan et al., 2024). Species allocating more N to roots may tend to thicken plant cell walls and showed better performance on the synthesis of defense substances to enhance plant resistance to herbivores and pathogens, resulting in high survival under conspecific neighbors. Our study reveals the interspecific variation in plant life-history trade-off mediated by the element allocation among different organs, whereas further studies may focus more on the chemical composition rather than element to dig the mechanism driving the plant life-history strategy.

## 5 Conclusion

In summary, combined data from an HSD 50-ha subtropical forest plot census, functional traits, and nutrient allocation among different organs showed that 1) N and P allocation between leaves and roots had significant interspecific variation compared with C allocation; 2) N allocation among leaves and roots determined interspecific PES, with species allocating more N in leaves always showing greater resource acquisition traits; and 3) fast-growing species experienced higher mortality rates with conspecific neighbors compared with slow-growing species, whereas species allocating more N to leaves showed higher growth rates and less sensitivity to conspecific neighbors. Our work demonstrated the interspecific variation in nutrient allocation among plant organs played a critical role in PES and the life history strategy trade-off.

## Data Availability

The original contributions presented in the study are included in the article/**Supplementary Material**. Further inquiries can be directed to the corresponding author.
