# Supplementary material for: Nitrogen allocation among leaves and roots mediates the interaction between plant life history trade-off and density dependence
Source: Front Plant Sci. 2025 Mar 12;16:1549801. doi: 10.3389/fpls.2025.1549801 (PMC11936912; doi:10.3389/fpls.2025.1549801)
Supplement: Supplementary file 1 [file DataSheet1.docx]

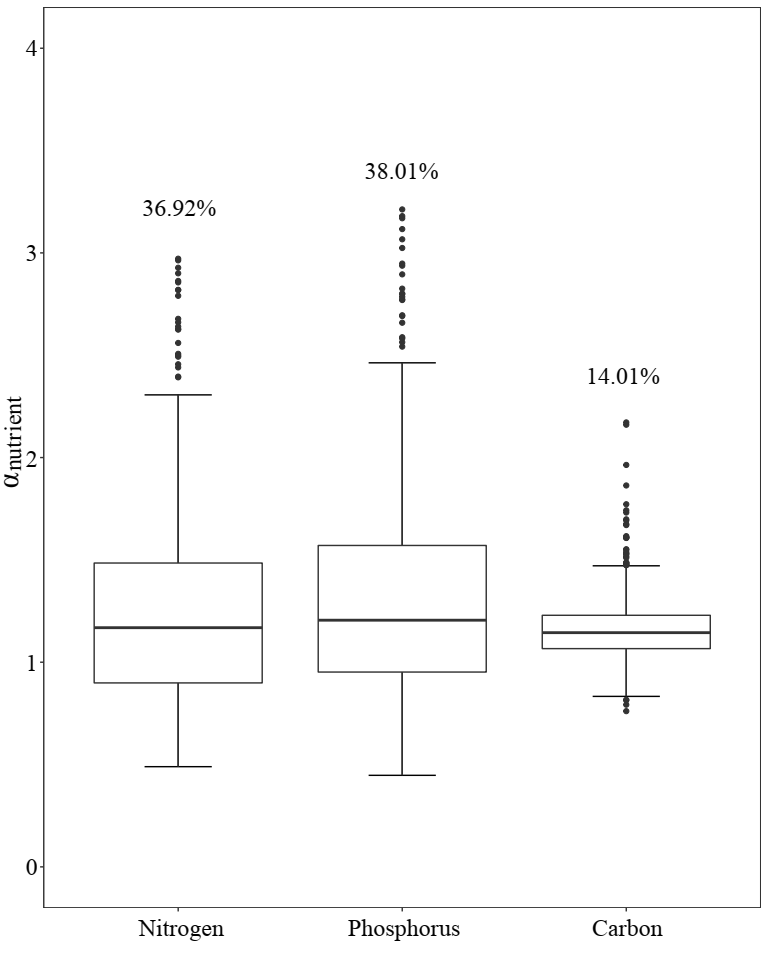


Fig. S1 Boxplot of leaf: root ratio of nitrogen, phosphorus and carbon of 92 woody species


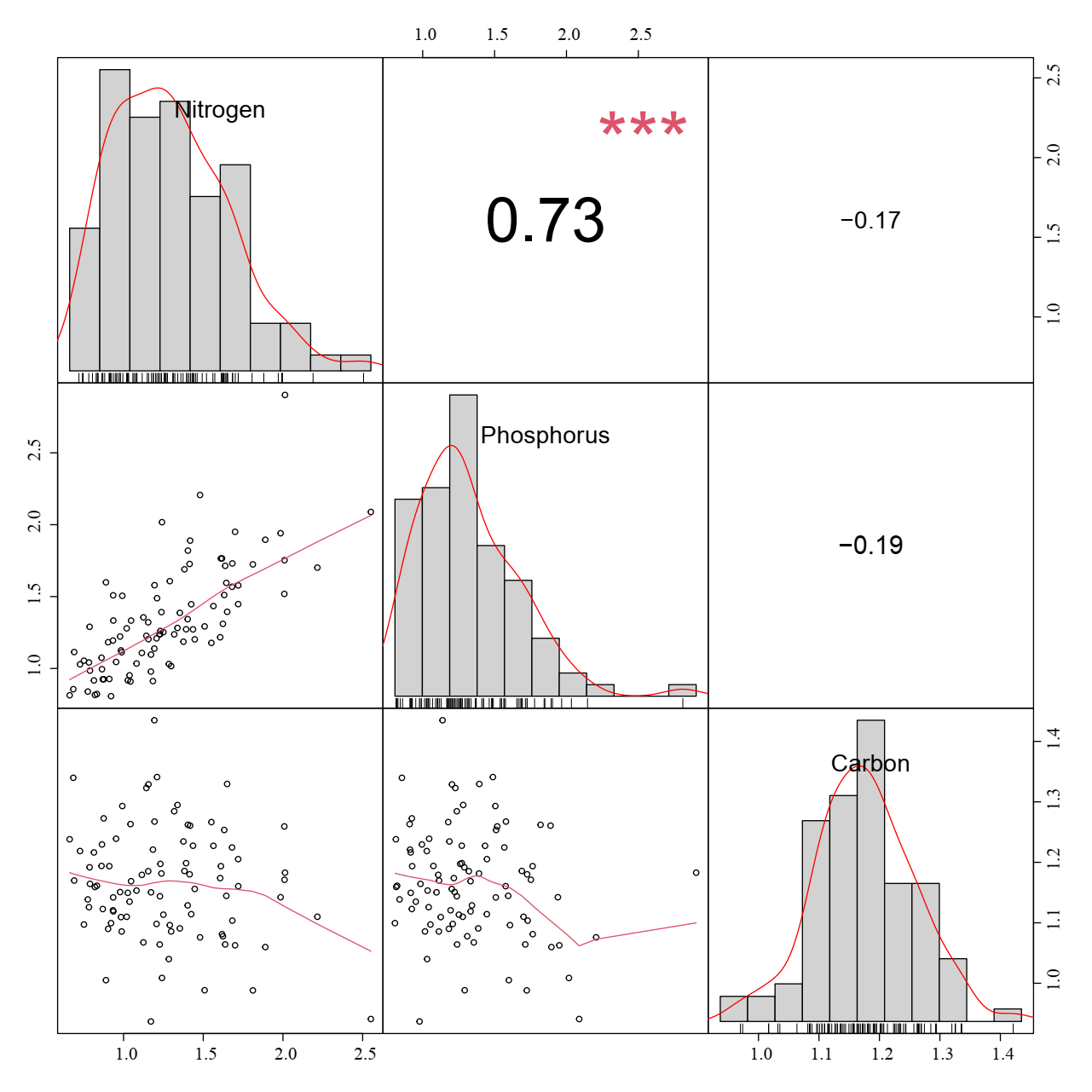


Fig. S2 Pairwise correlation between nutrient allocation between leaves and roots of 92 woody species. *** represents a significant relationship (*P* < 0.001) between nitrogen and phosphorus allocation among leaves and roots.


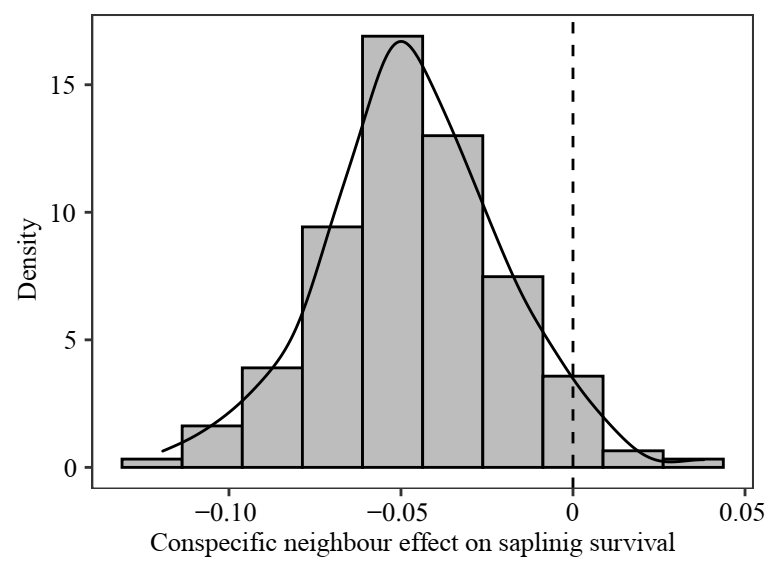


Fig. S3 Distribution of effects of conspecific neighbors on sapling survival. Histogram bars are based on posterior means of coefficients of coefficients for 92 woody species in Heishiding Nature Reserve. Bars to the left of the dashed zero line represent species whose survival is reduced by increasing density of conspecific neighbors.


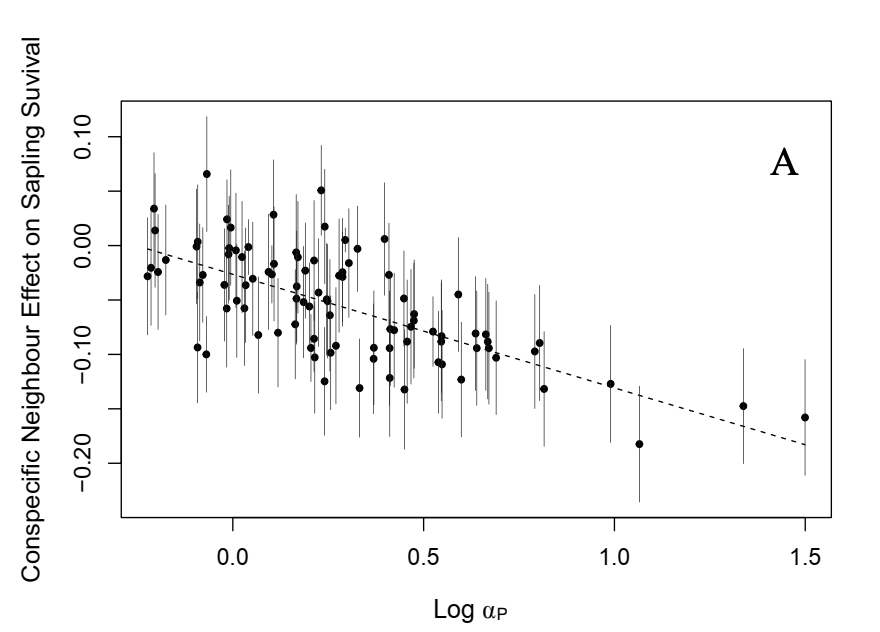


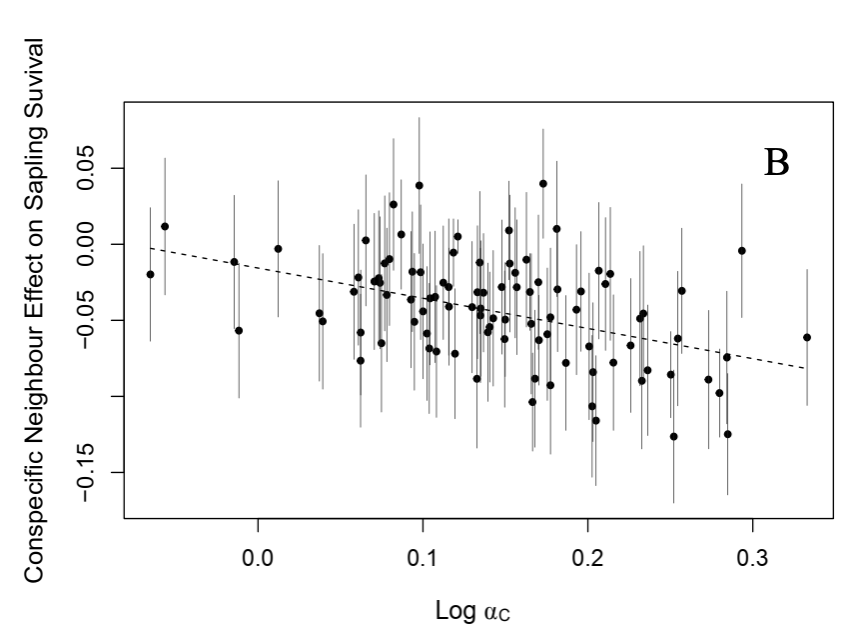


Fig. S4 Relationship between phosphorus (A) and carbon (B) allocation among leaves and roots and the species conspecific neighbor effects on sapling survival. Bars represent 95% confidence intervals. Dashed lines indicate non-significant relationship (*P* > 0.05).

Table S1 RGR95 and grouping of 92 woody species in Heishiding 50 ha dynamic plot

| Scientific.name | RGR95 | Groups |
| --- | --- | --- |
| Acer tutcheri | 0.0736 | slow-growing species |
| Altingia chinensis | 0.0748 | slow-growing species |
| Antidesma venosum | 0.0664 | slow-growing species |
| Artocarpus styracifolius | 0.0908 | median-growing species |
| Beilschmiedia fordii | 0.0622 | slow-growing species |
| Beilschmiedia tsangii | 0.1139 | median-growing species |
| Bridelia fordii | 0.2065 | fast-growing species |
| Canarium album | 0.1916 | fast-growing species |
| Castanopsis carlesii | 0.1795 | fast-growing species |
| Castanopsis eyrei | 0.0898 | median-growing species |
| Castanopsis fabri | 0.2051 | fast-growing species |
| Castanopsis fissa | 0.1769 | fast-growing species |
| Castanopsis fordii | 0.1342 | fast-growing species |
| Castanopsis hystrix | 0.095 | median-growing species |
| Castanopsis nigrescens | 0.1145 | median-growing species |
| Cinnamomum appelianum | 0.0605 | slow-growing species |
| Cinnamomum austrosinense | 0.0971 | median-growing species |
| Cinnamomum porrectum | 0.1936 | fast-growing species |
| Cinnamomum subavenium | 0.0996 | median-growing species |
| Cinnamomum validinerve | 0.0918 | median-growing species |
| Corylopsis multiflora | 0.0687 | slow-growing species |
| Cryptocarya chinensis | 0.1544 | fast-growing species |
| Cryptocarya concinna | 0.0972 | median-growing species |
| Cyclobalanopsis bambusaefolia | 0.1674 | fast-growing species |
| Cyclobalanopsis chungii | 0.0741 | slow-growing species |
| Cyclobalanopsis hui | 0.0995 | median-growing species |
| Diospyros morrisiana | 0.0873 | median-growing species |
| Diospyros strigosa | 0.0724 | slow-growing species |
| Diplospora dubia | 0.0464 | slow-growing species |
| Distylium racemosum | 0.0568 | slow-growing species |
| Elaeocarpus chinensis | 0.1315 | fast-growing species |
| Elaeocarpus decipiens | 0.1199 | fast-growing species |
| Engelhardtia fenzlii | 0.1316 | fast-growing species |
| Eurya acuminatissima | 0.0973 | median-growing species |
| Eurya hebeclados | 0.0883 | median-growing species |
| Eurya nitida | 0.083 | slow-growing species |
| Hartia villosa | 0.0739 | slow-growing species |
| Homalium cochinchinense | 0.0453 | slow-growing species |
| Ilex kwangtungensis | 0.0771 | slow-growing species |
| Ilex memecylifolia | 0.0816 | slow-growing species |
| Ilex subficoidea | 0.0601 | slow-growing species |
| Itea chinensis | 0.1352 | fast-growing species |
| Laurocerasus phaeosticta | 0.088 | median-growing species |
| Lindera chunii | 0.1033 | median-growing species |
| Lindera metcalfiana | 0.2401 | fast-growing species |
| Lithocarpus calophyllus | 0.1715 | fast-growing species |
| Lithocarpus haipinii | 0.1372 | fast-growing species |
| Lithocarpus litseifolius | 0.118 | fast-growing species |
| Lithocarpus lohangwu | 0.1093 | median-growing species |
| Lithocarpus uvariifolius | 0.0854 | median-growing species |
| Litsea acutivena | 0.1011 | median-growing species |
| Litsea greenmaniana | 0.0759 | slow-growing species |
| Litsea lancilimba | 0.0969 | median-growing species |
| Macaranga sampsonii | 0.0846 | slow-growing species |
| Machilus breviflora | 0.0767 | slow-growing species |
| Machilus chinensis | 0.0936 | median-growing species |
| Machilus litseifolia | 0.1251 | fast-growing species |
| Machilus velutina | 0.0912 | median-growing species |
| Manglietia fordiana | 0.0923 | median-growing species |
| Manglietia moto | 0.0809 | slow-growing species |
| Meliosma fordii | 0.085 | slow-growing species |
| Meliosma squamulata | 0.0689 | slow-growing species |
| Microtropis gracilipes | 0.0526 | slow-growing species |
| Mytilaria laosensis | 0.1046 | median-growing species |
| Neolitsea cambodiana | 0.1276 | fast-growing species |
| Neolitsea chuii | 0.1327 | fast-growing species |
| Neolitsea phanerophlebia | 0.146 | fast-growing species |
| Neolitsea pulchella | 0.1084 | median-growing species |
| Olea dioica | 0.0762 | slow-growing species |
| Ormosia glaberrima | 0.0668 | slow-growing species |
| Ormosia pachycarpa | 0.0701 | slow-growing species |
| Photinia prunifolia | 0.0906 | median-growing species |
| Polyalthia plagioneura | 0.2147 | fast-growing species |
| Rapanea neriifolia | 0.0806 | slow-growing species |
| Reevesia thyrsoidess | 0.1044 | median-growing species |
| Schefflera octophylla | 0.2451 | fast-growing species |
| Schima superba | 0.1228 | fast-growing species |
| Sinosideroxylon wightianum | 0.0942 | median-growing species |
| Sloanea sinensis | 0.1671 | fast-growing species |
| Styrax odoratissimus | 0.0642 | slow-growing species |
| Symplocos adenophylla | 0.1676 | fast-growing species |
| Symplocos anomala | 0.0872 | median-growing species |
| Symplocos cochinchinensis | 0.1298 | fast-growing species |
| Symplocos congesta | 0.1311 | fast-growing species |
| Symplocos lancifolia | 0.1154 | fast-growing species |
| Symplocos pseudobarberina | 0.176 | fast-growing species |
| Symplocos sumuntia | 0.0918 | median-growing species |
| Syzygium buxifolium | 0.0576 | slow-growing species |
| Syzygium kwangtungense | 0.075 | slow-growing species |
| Tutcheria championi | 0.0968 | median-growing species |
| Vitex quinata | 0.0973 | median-growing species |
| Xanthophyllum hainanense | 0.0629 | slow-growing species |

Table S2 AIC values for generalized linear models of sapling survival as a function of neighbor density, with different exponent ⍺ range from 0.1-1. The value of ⍺ that produced the lowest AIC value (i.e. the best fit to the data) is in bold. ∆AIC is the difference in AIC between the given model and the best fit model.

| Exponent ⍺ | AIC | ﻿∆AIC |
| --- | --- | --- |
| 0.1 | 102426.9 | 236.3 |
| 0.2 | 102375.9 | 185.3 |
| 0.3 | 102312.7 | 122.1 |
| 0.4 | 102249.5 | 58.9 |
| 0.5 | 102204 | 13.4 |
| **0.6** | **102190.6** | **0** |
| 0.7 | 102213.5 | 22.9 |
| 0.8 | 102265.8 | 75.2 |
| 0.9 | 102333.5 | 142.9 |
| 1 | 102403.3 | 212.7 |

Table S3 Phylogenetic signal of functional traits and nutrient allocation

| Functional traits | K | *P* |
| --- | --- | --- |
| LA | 0.109 | 0.324 |
| SLA | 0.213 | 0.003 |
| LDMC | 0.197 | 0.001 |
| T | 0.144 | 0.013 |
| DIAM | 0.332 | 0.001 |
| RBI | 0.304 | 0.001 |
| RTD | 0.303 | 0.001 |
| SRA | 0.187 | 0.001 |
| SRL | 0.266 | 0.001 |
| Nutrient allocation | K | *P* |
| ⍺_Nitrogen_ | 0.235 | 0.001 |
| ⍺_Phosphorus_ | 0.318 | 0.001 |
| ⍺_Carbon_ | 0.107 | 0.123 |
